# Efficiency of Portable Antennas for Detecting Passive Integrated Transponder Tags in Stream-Dwelling Salmonids

**DOI:** 10.1371/journal.pone.0149898

**Published:** 2016-02-22

**Authors:** Nolan P. Banish, Summer M. Burdick, Katherine R. Moyer

**Affiliations:** 1 U.S. Fish and Wildlife Service, Klamath Falls Fish and Wildlife Office, Klamath Falls, Oregon, United States of America; 2 U.S. Geological Survey, Western Fisheries Research Center, Klamath Falls Field Station, Klamath Falls, Oregon, United States of America; 3 Conservation and Land Management Internship Program, Chicago Botanic Garden, Glencoe, Illinois, United States of America; Pacific Northwest National Laboratory, UNITED STATES

## Abstract

Portable antennas have become an increasingly common technique for tracking fish marked with passive integrated transponder (PIT) tags. We used logistic regression to evaluate how species, fish length, and physical habitat characteristics influence portable antenna detection efficiency in stream-dwelling brown trout (*Salmo trutta*), bull trout (*Salvelinus confluentus*), and redband trout (*Oncorhynchus mykiss newberrii*) marked with 12-mm PIT tags. We redetected 56% (20/36) of brown trout, 34% (68/202) of bull trout, and 33% (20/61) of redband trout after a recovery period of 21 to 46 hours. Models indicate support for length and species and minor support for percent boulder, large woody debris, and percent cobble as parameters important for describing variation in detection efficiency, although 95% confidence intervals for estimates were large. The odds of detecting brown trout (1.5 ± 2.2 [mean ± SE]) are approximately four times as high as bull trout (0.4 ± 1.6) or redband trout (0.3 ± 1.8) and species-specific differences may be related to length. Our reported detection efficiency for brown trout falls within the range of other studies, but is the first reported for bull trout and redband trout. Portable antennas may be a relatively unbiased way of redetecting varying sizes of all three salmonid species.

## Introduction

The use of passive integrated transponder (PIT) tags in fisheries research has become widespread and has greatly advanced our understanding of fish behavior [1]. In monitoring studies, PIT tags are less expensive than other electronic tag types, require minimal handling of fish to obtain multiple detections, and last indefinitely. Multiple detections of fish are made possible with the use of flat plate or pass-through fixed antenna arrays positioned at discrete locations, such as at dams or spawning locations, enabling improved estimates of survival or other population parameters [2].

Increasingly, a combination of portable and fixed antennas are used to maximize PIT tag detections across a study area [3,4]. This is especially applicable to small streams where a single operator can scan the area of interest by carrying a transceiver, battery, and tuning mechanism connected to the antenna [5]. Portable antennas also have proven valuable for evaluating fish movement [6], improving rates of redetection [7], and for novel monitoring techniques, such as tracking tagged fish under ice covered streams [8]. However, to obtain reliable and unbiased estimates of population parameters, it is essential to account for variation in capture, or detection, efficiency [9].

Several studies have examined variation in detection efficiency of portable PIT tag antennas. Detection efficiency of portable antennas varies due to operator experience or technique [10,11] and is a function of tag size and transmission type (half or full-duplex; [12]). There is a negative relationship between detection efficiency and stream velocity or stage height [11,13,14], stream width [4], and water temperature [13]. Calmness of water and shadows cast on water also appear to influence detection efficiency [13]. Despite the emerging body of work evaluating the influence of physical habitat characteristics on detection efficiency, the effect of large woody debris has not been evaluated in fisheries applications.

Research to date has indicated that detection efficiency of portable antennas may be influenced by fish length, species, or a combination of the two. For example, 82% of small (72 mm mean length) brown trout (*Salmo trutta*) implanted with 12-mm full-duplex PIT tags were detected with a portable antenna, but only 69% of large (142 mm mean length) brown trout with 12-mm tags were detected [15]. Average detection efficiency for 12-mm full-duplex PIT tags reported in published studies varies dramatically among species [10]. However, differences in detection efficiency among species may be explained by fish length. [15] reported the same detection efficiency for slimy sculpin (*Cottus cognatus*) and brown trout, when they controlled for stream, fish length, and tag type.

Here, our intent was to evaluate the influence of biotic (species and length) and abiotic variables on portable antenna detection efficiency for stream-dwelling salmonids tagged with 12-mm full-duplex PIT tags. We were specifically interested in 1) evaluating how detection efficiency varies by size and among PIT-tagged brown trout, bull trout (*Salvelinus confluentus*), and redband trout (*Oncorhynchus mykiss newberrii*); and 2) evaluating how physical characteristics of study areas influence detection efficiency. We report the first evaluation of portable antenna detection efficiency for PIT-tagged bull trout and redband trout and this work furthers our understanding of portable antenna efficiency by assessing the effects of large woody debris and other habitat variables.

## Methods

### Study Area

We evaluated portable antenna efficiency at Leonard Creek, Brownsworth Creek, and Boulder Creek in the upper Klamath River basin located in Klamath and Lake Counties, south central Oregon, USA (42°30ʹN, 120°51ʹW). Leonard Creek is a tributary to Brownsworth Creek, which flows into the South Fork Sprague River, and Boulder Creek is a tributary to the North Fork Sprague River. These creeks were chosen for this study since they are the only streams in the Klamath River basin that contain brown trout, bull trout, and redband trout. Each creek is a first or second order stream (based on a 1:24,000 U. S. Geological Survey topographic map) that originates in the Gearhart Mountain Wilderness. Land ownership in these drainages is U. S. Forest Service at the headwaters and private timberland downstream. The hydrograph of these creeks is representative of a spring snowmelt pattern. Reaches varied between 1671 m and 1727 m in elevation. All sampling occurred during July 2012 when discharge was at or near summer base flow. We did not determine discharge at Brownsworth Creek or Boulder Creek during the course of sampling; however, discharge at Leonard Creek was calculated to be 0.08 m^3^/s using a cross sectional method [16].

### Field Survey Methods

Within each of the three creeks, we aimed to establish three enclosed reaches that were 250 m in length. Actual distances varied based on suitability of block net placement (range 164 m to 445 m; Table 1). Beginning at the downstream end of each reach, we made one upstream pass with a backpack electrofishing unit (Smith-Root model LR-24; Vancouver, Washington, USA) set at 650 to 750 V, 50 to 60 Hz, and pulsed direct current, to capture trout in each creek. All captured trout were anesthetized using tricaine methanesulfonate (MS-222) and fork length (FL) measured to the nearest millimeter. Trout ≥ 100 mm were tagged with a 12-mm × 2.15-mm full-duplex PIT tag (0.1 g, 134.2 kHz; Oregon RFID, Portland, Oregon, USA) using a 12-gauge needle inserted into the dorsal musculature of the fish posterior to the dorsal fin. As part of a concurrent study, previously PIT-tagged trout (≥ 100 mm) occurred within some of the reaches at Leonard and Brownsworth creeks. Any previously tagged trout that were captured were included in this study. We did not tag any trout < 100 mm to minimize potential for injury and to capitalize on the tagged trout already present in Leonard and Brownsworth creeks. After handling and tagging, trout were placed in a bucket of fresh creek water until they regained equilibrium. Trout were subsequently released evenly throughout the enclosed reach from which they were captured.

**Table 1:** Physical habitat characteristics (mean ± SD) in Leonard, Brownsworth, and Boulder creeks (Oregon, USA).

Variable	Leonard	Brownsworth	Boulder
Mean reach length (m)	254.6 (64.7)	316.5 (96.4)	250.0 (11.4)
Mean wetted width (m)	2.33 (0.04)	2.17 (0.16)	2.66 (0.35)
Percent undercut bank	12.9 (4.1)	11.1 (1.2)	6.0 (1.4)
Percent pool	13.3 (2.6)	15.8 (0.9)	24.4 (3.8)
LWD density (pieces/m^2^)	0.03 (0.008)	0.03 (0.006)	0.05 (0.017)
Percent cobble	16.7 (6.2)	25.0 (0.0)	36.7 (20.9)
Percent boulder	5.0 (4.1)	5.0 (0.0)	30.0 (14.7)

LWD, large woody debris

This study was carried out with approval and in strict accordance with the terms and conditions outlined under Section 10(a)(1)(A) of the Endangered Species Act federal recovery permits (permit FWSKFFWO-7 and TE-108507) and the state of Oregon scientific taking permit (permit number: 17206). Both federal and state permits allowed the capture, handling, anesthetization, and surgery (marking) performed on the fish in this study. All surgery was performed under MS-222 and all efforts were made to minimize suffering. No fish that exhibited stress were subject to surgery.We allowed marked fish to recover 21 to 46 hours after electrofishing before returning to survey with the portable antenna. A 24-hour recovery and dispersal period has been shown to be long enough such that marking and handling do not affect detection efficiency [17]. To determine portable antenna detection efficiency, one upstream pass was made through each reach using a Biomark BP portable antenna (Boise, Idaho, USA). The portable antenna was connected to a chest pack containing a Biomark portable transceiver (model FS2001FS-ISO) that recorded all detected PIT tags, a tuning box that controlled antenna current, and a 12-V sealed lead acid battery that provided power. In field tests, the portable antenna was capable of detecting 12-mm PIT tags oriented perpendicular to the antenna face at distances up to 35 cm. Four surveyors with equal experience took turns operating the portable antenna. Surveyors operated the portable antenna in a systematic manner to ensure all habitats were scanned. The time spent operating the portable antenna within each reach was 63.6 ± 11.3 min (mean ± SD). All surveys were conducted during day light hours and block nets were removed upon completion of surveying.

We recorded several habitat variables at each reach to determine how stream complexity influences portable antenna efficiency. Beginning at the downstream end of each reach, we established transects perpendicular to the flow approximately every 25 m. At each transect, we recorded wetted width and visually estimated cobble (64 to 256 mm) and boulder (256 to 4,096 mm) substrate to the nearest 5% along a 1-m wide band centered on the transect. In each reach, we summed length of undercut banks and conveyed this as a percent of total bank length (left and right). An undercut bank was defined as at least 15.2 cm of undercut and 30.5 cm long, which we assumed to provide cover for fish. We summed the number of large woody debris pieces and calculated the density within each reach. Large woody debris was defined as any piece of wood at least 3 m long by 10 cm in diameter located sufficiently within the wetted channel such that it was capable of providing cover. The length of each pool was measured throughout each reach, summed and multiplied by average wetted width, and expressed as the percentage of pool habitat for the entire reach. Only pools that spanned the entire wetted width were measured.

### Data Analyses and Statistics

To evaluate portable antenna efficiency, we developed an a priori suite of logistic regression models (hypotheses) using detection of PIT-tagged trout as a binary response variable (detected = 1, not detected = 0). Efficiency was defined as the probability of detecting a fish. We did not run all possible combinations of models since we selected models based on specific biological hypotheses. Previous studies have reported detection efficiency may vary by fish species, size, and physical habitat characteristics [17,18]. Thus, fish characteristics (species and length), physical habitat characteristics (percent bolder, percent cobble, number of pieces of large woody debris; Table 1), and stream were included as model predictors to evaluate portable antenna efficiency. We also included individual operator and time spent surveying (minutes per unit length) to confirm that surveys were conducted in a consistent manner. Continuous variables were converted to proportions to allow for ease of comparison. A null (dot) model also was included to represent no biotic or abiotic influence on detection efficiency.

Prior to model development, we checked for correlation between continuous variables using Pearson product-moment correlation coefficients with the ‘rcorr’ function (package Hmisc) in program R, version 2.15 [19]. Variables were considered highly correlated when *r*^2^ values were greater than 0.5 [20]. The proportion of pool habitat was positively correlated with proportion of boulder substrate and large woody debris was positively correlated with the proportion of undercut banks. Therefore, we did not consider the proportion of pool habitat or undercut banks in our model set as large woody debris has not been evaluated previously and we believed proportion of boulder substrate to provide more suitable fish cover. We also checked for correlation between categorical variables using a polyserial correlation conducted with the ‘polyserial’ function (package polycor) and identified a slight correlation (*r*^2^ = 0.16) between species and length [21]. Brown trout were slightly larger (166 ± 30 mm FL [mean ± SD]) on average than the other two species (bull trout, 130 ± 26 mm FL; redband trout, 124 ± 25 mm FL). Therefore, we considered a model with just length, a model with just species, an additive model with both (three intercepts and one slope), and a model with a species by length interaction (three intercepts and three slopes) to examine differences in detection efficiency among species and lengths.

We assessed the goodness of fit of a model containing all the variables of interest (global model) by estimating the variance inflation factor (ĉ). A ĉ of 1.0 indicates a perfect fit and a ĉ over 4.0 indicates overdispersion [22]. We ranked models based on the principle of parsimony with Akaike’s Information Criteria adjusted for small sample size (AICc; [22]) using the logLik function in the ‘MASS’ package [23]. To avoid redundancy, we do not report ĉ for models with a lower AICc value than the global model; these models were better fit to the data than the global model. We considered models with AICc values no more than 2.0 units greater than the lowest AICc to be well supported [22] and included these models in our confidence set. Normalized model weights (*w*_*i*_) were calculated to determine the probability that a model was the best one in the set for explaining the data. Importance weights were calculated as the sum of model weights containing a parameter of interest [24]. We generated model-averaged parameter estimates and unconditional 95% confidence intervals for all continuous variables in our confidence set of models. Lastly, we calculated the odds of detecting each species and report the unconditional standard errors for these odds [25]. Unconditional confidence intervals and standard errors account for uncertainty in both model fit and model selection, and are therefore larger than standard errors conditioned on any single model being the true model.

## Results

**Fig 1:**
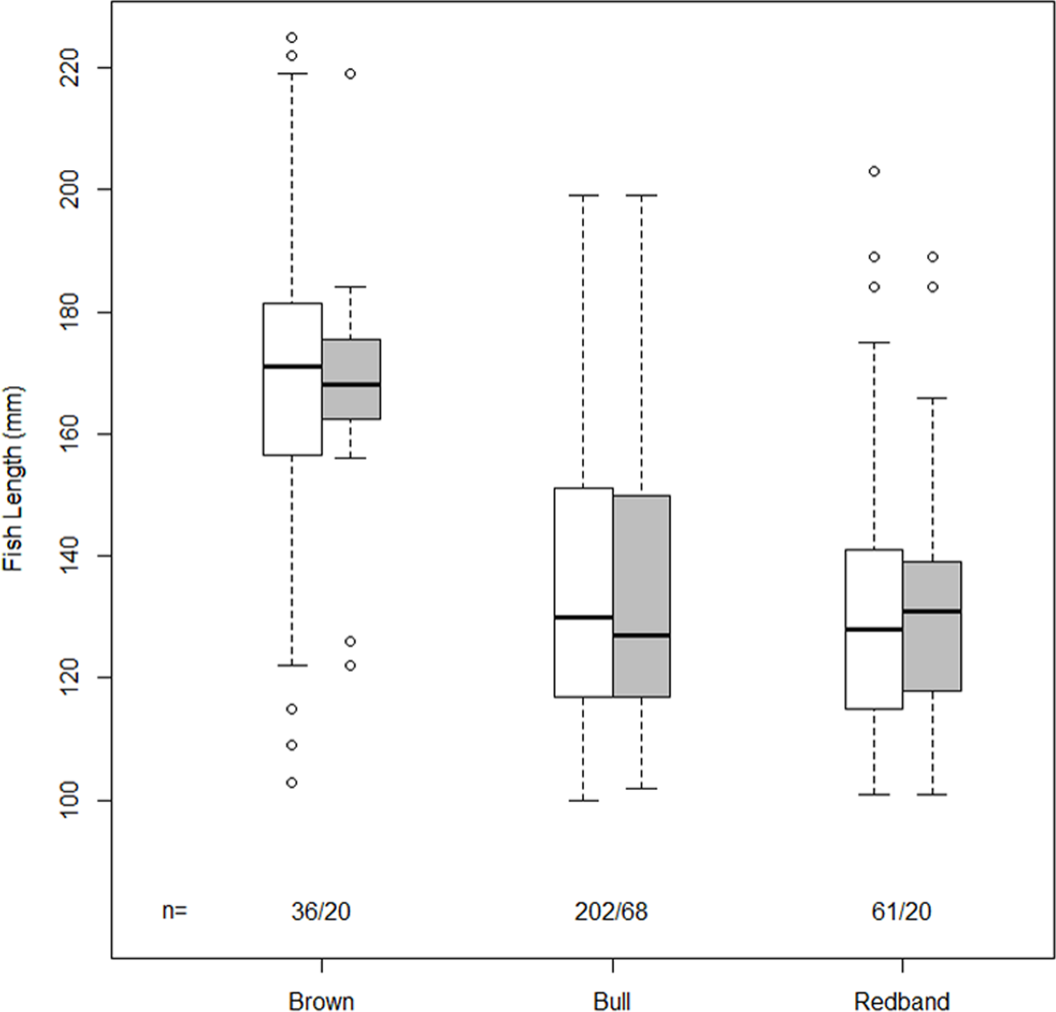
Box plots of fish length (mm) for all PIT-tagged brown trout, bull trout, and redband trout in Leonard Creek, Brownsworth Creek, and Boulder Creek (white box) and for those individuals that were redetected (gray box). The lower and upper boundaries of each box indicate the 25th and 75th percentiles, respectively, and the median value is shown as a line within the box. The lines (whiskers) below and above each box represent the 10th and 90th percentiles. All outliers are presented.

We physically captured 36 brown trout, 202 bull trout, and 61 redband trout that were given new PIT tags (91% of captures) or were previously PIT tagged (9% of captures) in Leonard, Brownsworth, and Boulder creeks during the course of the study. Brown trout ranged from 103 mm to 225 mm FL, bull trout from 100 mm to 199 mm FL, and redband trout from 101 mm to 203 mm FL. Overall, we redetected 56% (20/36) of PIT-tagged brown trout, 34% (68/202) of bull trout, and 33% (20/61) of redband trout. Box plots revealed that the lengths of each species of fish that were redetected closely matched lengths of all fish that were marked in this study (Fig 1).

Our goodness of fit test revealed a ĉ of 1.3, demonstrating the global model fit the data well. Model rankings indicated that five explanatory variables had some effect on detection efficiency (Table 2). Our confidence set contained six models; of these, three contained length, three contained species, and one each contained percent boulder, large woody debris, and percent cobble. Length carried an importance weight of 0.50 and species carried an importance weight of 0.44, whereas percent boulder, large woody debris, and percent cobble, carried importance weights of 0.32, 0.20, and 0.13, respectively. The top ranked model indicated that larger fish were easier to detect when boulder cover was scarce, but was only slightly more parsimonious than other models in the confidence set. Model rankings provided scarce evidence that portable antenna operator, time spent surveying, and stream had an effect on detection efficiency.

**Table 2:** Model ranking statistics for models describing factors influencing portable antenna detection efficiency.

Model	*K*	Log likelihood	AICc	ΔAICc	*w*_*i*_
LENGTH * BOULDER	**4**	**-190.82**	**389.77**	**0.00**	**0.19**
**SPECIES + LARGE WOODY DEBRIS**	**4**	**-190.94**	**390.02**	**0.25**	**0.17**
**LENGTH**	**2**	**-193.34**	**390.72**	**0.95**	**0.12**
**SPECIES**	**3**	**-192.36**	**390.79**	**1.03**	**0.12**
LENGTH * COBBLE	**4**	**-191.52**	**391.18**	**1.41**	**0.09**
**SPECIES + LENGTH**	**4**	**-191.71**	**391.56**	**1.79**	**0.08**
SPECIES + BOULDER	4	-191.89	391.92	2.15	0.07
COBBLE + BOULDER	3	-193.50	393.08	3.32	0.04
DOT	1	-195.58	393.17	3.40	0.04
LARGE WOODY DEBRIS	2	-195.07	394.17	4.41	0.02
OPERATOR	5	-192.08	394.36	4.59	0.02
SPECIES * LENGTH	6	-191.29	394.87	5.11	0.01
BOULDER	2	-195.54	395.11	5.35	0.01
SURVEY TIME	3	-194.98	396.04	6.27	0.01
BOULDER + LARGE WOODY DEBRIS	3	-194.98	396.05	6.28	0.01
STREAM	3	-195.27	396.62	6.85	0.01
GLOBAL	12	-188.18	401.45	11.69	0.00

Note: Models in bold indicate the confidence set of models.

**Fig 2:**
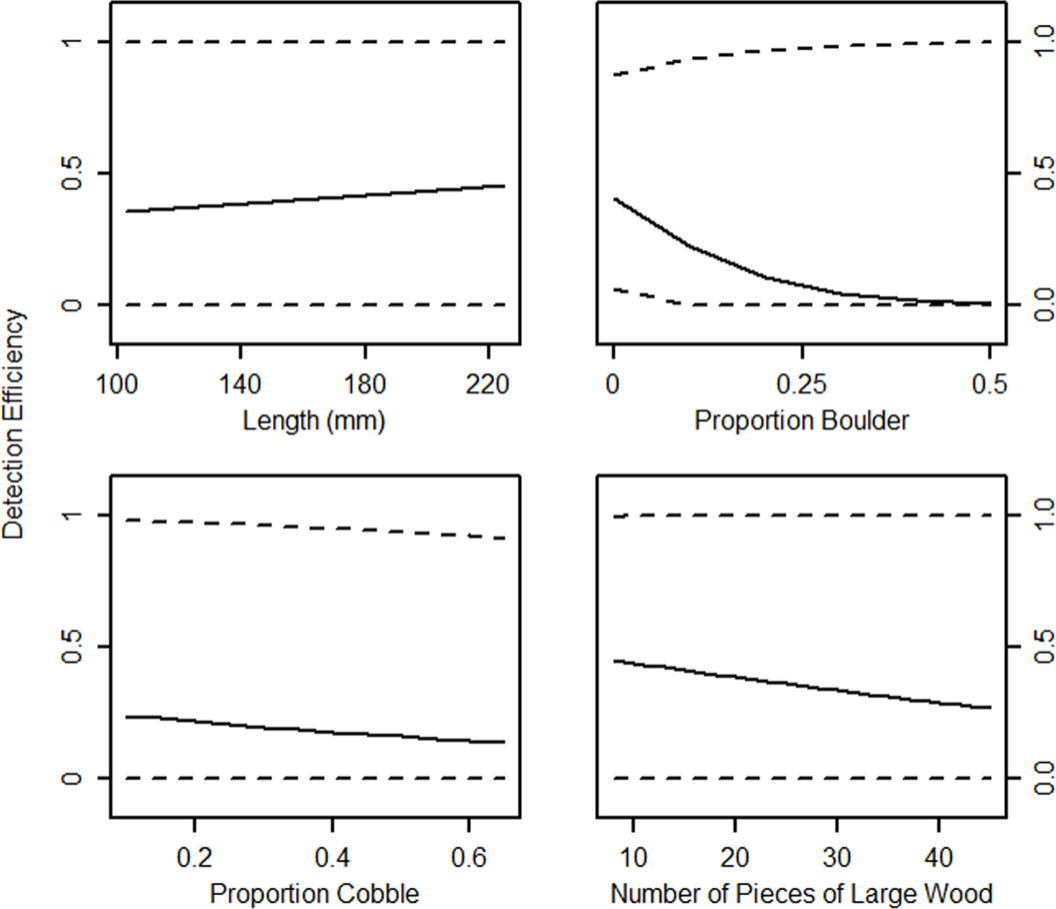
Model averaged estimates of portable antenna detection efficiency for PIT-tagged brown trout, bull trout, and redband trout in Leonard Creek, Brownsworth Creek, and Boulder Creek. Model averaged unconditional 95% confidence intervals for estimates are displayed as dotted lines.

There was a wide distribution in the normalized model weights among models that ranked higher than the null (dot) model, indicating relatively little support for any of the hypotheses fit to the data. Length was positively correlated with detection efficiency, whereas proportion of boulder substrate, proportion of cobble substrate, and large woody debris were negatively correlated with detection efficiency (Fig 2). However, 95% confidence intervals for estimates were wide, indicating that these effects were not strongly supported (Fig 2). The odds (± SE) of detecting brown trout (1.5 ± 2.2), bull trout (0.4 ± 1.6), and redband trout (0.3 ± 1.8) were imprecisely estimated (i.e., large standard errors). Nevertheless, odds indicated brown trout were on average four times as likely to be detected as bull trout or redband trout. Model rankings indicated there was more likely to be an additive, rather than interactive, effect of length and species. The additive length and species model indicated estimated detection efficiency ranged from 46% to 64% for brown trout, 29% to 47% for bull trout, and 30% to 48% for redband trout within the range of fish lengths in this study (100 mm to 225 mm FL).

## Discussion

Our estimates of detection efficiency for brown trout fall within the range of other studies examining the detection efficiency of portable antennas for brown trout tagged with 12-mm PIT tags. Mean brown trout detection efficiency has been reported at 43% for fish with mean FL 119 mm [10] and at 69% for fish with mean FL 148 mm [15]. Based on our additive species and length model, the estimated detection efficiency (conditional 95% confidence interval) is 49% (4% to 95%) for a 119 mm FL brown trout and 53% (4% to 97%) for a 148 mm FL brown trout. There are no previous studies specifically examining portable antenna detection efficiency of bull trout or redband trout. Our results indicated detection efficiency for these two species was lower than brown trout. Our detection efficiency was estimated to be 32% (6% to 78%) for a 119 mm FL bull trout and 32% (5% to 81%) for a 119 mm FL redband trout.

Our results indicate that portable antennas may be a relatively unbiased way of redetecting varying sizes (100 to 225 mm in this study) of all three salmonid species under the physical habitat characteristics encountered in our study streams. A wide distribution in normalized model weights in the top set of models could either indicate that these models were capturing similar components of the variation in the data or that hypothesized length, species, and habitat effects on detection efficiency were minor. All of the top seven models had either a length or a species effect, indicating that these two variables explained nearly all of the variation in detection efficiency. When all models that included length or species were compared collectively to the dot model, each of these variables explained approximately four times the variation in detection efficiency as the dot model. However, the effect sizes of length and species were imprecisely estimated indicating that these effects were minor. Future research may be needed to provide additional information how these effects apply to fish < 100 mm in length.

The effects of the physical habitat covariates on portable antenna detection efficiency were minor to undetectable and unconditional 95% confidence intervals were large. Despite the influence of the physical habitat, the box plots indicated that there was concordance between the length of each species of fish that we redetected and the lengths of all fish that were initially PIT-tagged. Similar findings also have been reported for shorthead sculpin (*Cottus confusus*), Coho salmon (*O*. *kisutch*), and steelhead (*O*. *mykiss*) [26,27].

Differences in detection among species may be related to fish length. Species and length had nearly identical importance weights indicating that both of these variables explained about the same amount of variation in detection efficiency. Including models with additive or interactive effects of these two variables provided no additional insight because these more complicated models ranked lower than both of the single variable models. Therefore, despite length and species being only slightly correlated (*r*^2^ = 0.16), we cannot distinguish which of these variables is causing the majority of the variation in detection efficiency.

Alternatively, differences in detection efficiency among species may be related to behavior, which can be differentially influenced by environmental conditions. [10] speculated that species with a propensity to hide were easier to detect whereas those with a propensity to flee were harder to detect. Previous research has demonstrated that electrofishing and snorkeling within streams may cause salmonids, including bull trout and rainbow trout (*O*. *mykiss*), to flee in response to sampling [28,29]. Similarly, redband trout and bull trout may have fled as an operator approached with a portable antenna, resulting in lower detection efficiency of these two species compared to brown trout.

Further, the reaction of a fish to an approaching antenna may be related to the environment. For example, [13] determined that the flight response of Atlantic salmon (*Salmo salar*) surveyed with a portable antenna was influenced by water temperature. The three species in our study have different, but overlapping, temperature tolerances [30], and may react differently to the same disturbance given the ambient conditions. Species-specific diurnal activity patterns also might affect detection efficiency. For instance, bull trout appear to be more docile and easily captured by netting at night compared to during the day [31]. As well, portable antenna detection efficiency for Atlantic salmon is higher at night than during the day [11], but there is no difference between day and night detection rates of brown trout [10]. Future research may be required to determine if day or nighttime surveys are more effective for bull trout and redband trout.

We have demonstrated the effectiveness of using portable antennas for detecting three species of PIT tagged salmonids in small stream reaches (mean wetted width < 3.0 m) within a reasonable amount of time (63.6 ± 11.3 min [mean ± SD]). The lack of physical habitat effects within the range of habitats encountered in our study and the minor role of species and length on detection probability make it possible to use portable antennas in mark-recapture studies of stream-dwelling salmonids to estimate a wide variety of population parameters. Such information will prove to be useful when designing studies on vital rates of these trout species.
